# Regional variation in practitioner employment in general practices in England: a comparative analysis

**DOI:** 10.3399/bjgp20X708185

**Published:** 2020-02-11

**Authors:** Sharon Spooner, Jon Gibson, Kath Checkland, Anne McBride, Damian E Hodgson, Mark Hann, Imelda McDermott, Matt Sutton

**Affiliations:** Centre for Primary Care Research, School of Health Sciences, Faculty of Biology, Medicine and Health, University of Manchester, Manchester.; Centre for Primary Care Research, School of Health Sciences, Faculty of Biology, Medicine and Health, University of Manchester, Manchester.; Centre for Primary Care Research, School of Health Sciences, Faculty of Biology, Medicine and Health, University of Manchester, Manchester.; Alliance Manchester Business School, University of Manchester, Manchester.; Alliance Manchester Business School, University of Manchester, Manchester.; Centre for Primary Care Research, School of Health Sciences, Faculty of Biology, Medicine and Health, University of Manchester, Manchester.; Centre for Primary Care Research, School of Health Sciences, Faculty of Biology, Medicine and Health, University of Manchester, Manchester.; Centre for Primary Care Research, School of Health Sciences, Faculty of Biology, Medicine and Health, University of Manchester, Manchester.

**Keywords:** employment, general practice, health workforce, primary care networks, statistics and numerical data

## Abstract

**Background:**

In recent years, UK health policy makers have responded to a GP shortage by introducing measures to support increased healthcare delivery by practitioners from a wider range of backgrounds.

**Aim:**

To ascertain the composition of the primary care workforce in England at a time when policy changes affecting deployment of different practitioner types are being introduced.

**Design and setting:**

This study was a comparative analysis of workforce data reported to NHS Digital by GP practices in England.

**Method:**

Statistics are reported using practice-level data from the NHS Digital June 2019 data extract. Because of the role played by Health Education England (HEE) in training and increasing the skills of a healthcare workforce that meets the needs of each region, the analysis compares average workforce composition across the 13 HEE regions in England

**Results:**

The workforce participation in terms of full-time equivalent of each staff group across HEE regions demonstrates regional variation. Differences persist when expressed as mean full-time equivalent per thousand patients. Despite policy changes, most workers are employed in long-established primary care roles, with only a small proportion of newer types of practitioner, such as pharmacists, paramedics, physiotherapists, and physician associates.

**Conclusion:**

This study provides analysis of a more detailed and complete primary care workforce dataset than has previously been available in England. In describing the workforce composition at this time, the study provides a foundation for future comparative analyses of changing practitioner deployment before the introduction of primary care networks, and for evaluating outcomes and costs that may be associated with these changes.

## INTRODUCTION

The foundation for comprehensive health services provision is cost-effective universal primary care.^1^^–^3 However, many countries report problems with providing adequate access to these services^4^^–^6 because of difficulties with recruitment and retention of doctors trained to provide community-based generalist health care.^7^^–^9 Health policy has therefore focused on addressing the shortfall in workforce capacity and associated problems.^10^^–^12 Variation in primary care resources and expectations across different health systems (including funding, roles, and workload in general practice) makes it difficult to interpret differences in workforce composition between countries.^13^ In addition, there are limited data about the extent and impact of care provided by non-physicians.^14^^,^^15^ Birch *et al*’s extended analytical framework,^16^ which aligns human resource planning with population needs and provider characteristics, recognises that there are other factors that make it difficult to estimate healthcare need and make adequate investment in workforce training.^17^ This article addresses these issues in the context of the English NHS.

### Context of UK primary care workforce

While the number of practising doctors (per 100 000 population) in the UK is lower than in many European countries,^18^ the headcount of GPs (79.57 per 100 000 population in 2013) is close to the mean European Union level (79.47 per 100 000 population; range 9.12–160.11).^15^ Workforce modelling indicates a continuing projected shortage of GPs and practice nurses.^19^^–^21 An insufficient number of GPs will be available as a result of historic recruitment deficits and poor career retention,^22^^–^25 and there has been little increase in the number of nurses.^26^

Increasing workloads have led to government policy changes including recent recommendations regarding the deployment of a broader range of practitioner types.^27^^,^^28^ This is often termed ‘skill mix’ and it is proposed that a wider range of practitioner skills in the workforce, such as physiotherapists, paramedics, physician associates, pharmacists, and advanced nurse practitioners, should in future provide better alignment with projected healthcare needs.^29^^,^^30^ In addition to diversification within GP practices, from July 2019, structural and funding changes have begun to facilitate employment of practitioners to deliver integrated out-of-hospital care by working across more than one GP practice through the formation of primary care networks (PCNs).^27^^,^^31^

This article reports an analysis of new data that describe the composition of the English primary care workforce immediately before an anticipated workforce expansion associated with the introduction of PCNs. Following the lead of previously published analyses of the geographical distribution of GPs and practice nurses,^32^^,^^33^ this study looked at regional differences in workforce composition. In this study, workforce composition was compared using the 13 Health Education England (HEE) regions as geographical units, since the previously used administrative boundaries no longer exist or have relevance for decisions about staff employment. This choice also recognises the pivotal role played by HEE in workforce planning, training, and commissioning in response to local needs and changing workforce requirements.^31^

**Table:** How this fits in

Previous analyses of primary care workforce data have lacked access to the detailed information about newer types of practitioners that is now available. This study describes baseline employment patterns against which future changes can be assessed and facilitates analyses to consider associations with health outcomes and costs. Results of the study indicate that GPs and practice nurses significantly outnumber other practitioner groups in the primary care workforce, and few newer types of practitioner are reported. Comparison of practitioner deployment across Health Education England regions highlights differences that may be associated with regional variation in workforce planning, training, and commissioning.

NHS Digital routinely gathers data about the primary care workforce in England and publishes quarterly reports of employment across different practitioner types. A comprehensive analysis of these data was undertaken to provide a detailed picture of the location and work participation using full-time equivalent (FTE) of all practitioner types and to identify shifts in the proportion of practitioner types in the workforce.

This study aimed to examine NHS Digital data for variations in practitioner employment using richer data than have previously been available; to provide a baseline analysis of the workforce composition before any impact of PCNs; to look for geographical variation that may be associated with historical employment and/or HEE-prioritised activity; and to set out a methodological basis for identification of associations between workforce composition and data about healthcare activity and quality, and for establishing how progressive changes may be associated with health outcomes and costs.

## METHOD

### Data

The study uses practice-level workforce data that are publicly available from NHS Digital as part of the Workforce Minimum Data Set. This is a quarterly extraction of data that GP practices are contractually required to provide about staff working at NHS GP practices or other primary care organisations in England. Detailed guidance about the reporting requirements are provided online.^34^ Statistics are reported using the practice-level data from 30 June 2019 data extract.

### Categorisation of workers

Data are split across four workforce groups: GPs, nurses, direct patient care, and administration. Administration roles are omitted from this study because the focus is on workers who deliver patient care.

There are four categories of GP: GP partners, salaried GPs, locum GPs, and doctors training as GPs.

From the nursing categories, statistics are presented for practice nurses and advanced nurses, which is a composite category, constructed because of issues with role descriptors and low numbers in some constituent roles. The category is defined as the sum of the advanced, specialised, and extended role nursing categories. Because of their low level in reported data, trainee nurses are omitted from this analysis.

From the direct patient care group, the study includes healthcare assistants, pharmacists, physiotherapists, physician associates, and paramedics (roles directly responsible for healthcare delivery).

### Level of analysis

Workforce statistics are presented for each of the 13 HEE regions because of the multiple levels at which HEE activity may have an impact on the local availability of primary care practitioners.^31^

### Statistics

For each HEE region and staff group the study presents:

- the proportion of practices in the region who employ some of the staff group;
- total headcount of each staff group in the region;
- total FTE of each staff group in the region; and
- the mean FTE per thousand patients for each staff group in the region.

Practices with <1000 patients are omitted because these practices are opening, closing, or serving special populations, and are therefore atypical.

## RESULTS

**Figure 1.**
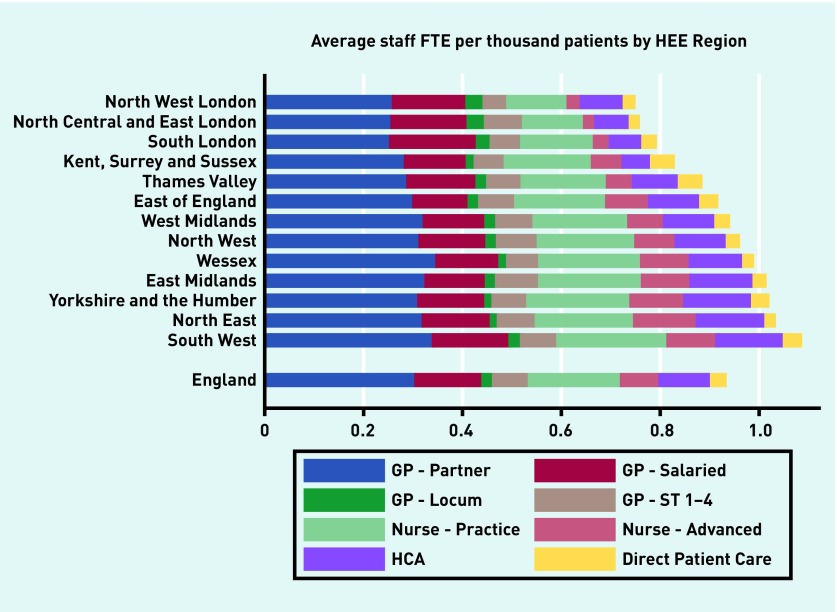
***Average full-time equivalent (FTE) staff per thousand patients by Health Education England (HEE) region. HCA = healthcare assistant.***

Table 1 shows the numbers of practices in each region and list size while Figure 1 summarises regional employment. A total of 86 practices with missing or atypical characteristics (as indicated by having <1000 registered patients or a missing list size), were excluded from this analysis. Substantial variability is noted in the number of omitted practices across the HEE regions and the average patient list size varies from 7119 in North West London to 11 272 in Wessex, while the mean list size for England is 8775 patients.

**Table 1.** Practice list size statistics for each Health Education England region

**HEE Region**	**Practices**	**Practices dropped for missing list size or <1000 patients**	**Practices in analysis**	**Mean list size**	**SD list size**
East Midlands	543	4	539	9046.98	5478.40
East of England	681	16	665	9872.38	6267.91
Kent, Surrey and Sussex	508	4	504	9641.87	5254.84
North Central and East London	490	5	485	8004.37	4229.52
North East	336	2	334	8327.72	4886.66
North West	1080	12	1068	7344.77	5065.17
North West London	360	4	356	7119.47	4575.85
South London	405	7	398	9265.17	5847.61
South West	506	5	501	10045.48	5947.01
Thames Valley	243	4	239	11071.21	5573.64
Wessex	262	3	259	11271.80	6353.33
West Midlands	803	9	794	7886.93	5816.66
Yorkshire and the Humber	683	11	672	8780.64	6184.08
England	6900	86	6814	8774.85	5659.74

HEE = Health Education England. SD = standard deviation.

Table 2 presents workforce statistics for GPs in each HEE region. A total of 92.38% of practices report having at least one GP partner, with 67.92% employing at least one salaried GP. The total FTE to total headcount ratio is lower among salaried GPs than partner GPs; the average FTE for partner GPs is 0.85 and for salaried GPs is 0.63 (totals not shown in table).

**Table 2.** Workforce statistics for GPs in each Health Education England region

**HEE Region**	**Partner**				**Salaried**				**Locum**				**ST 1–4**			
	**Practice with HC> 0, %**	**Total HC**	**Total FTE**	**Mean FTE PTP**	**Practice with HC> 0, %**	**Total HC**	**Total FTE**	**Mean FTE PTP**	**Practice with HC> 0, %**	**Total HC**	**Total FTE**	**Mean FTE PTP**	**Practice with HC> 0, %**	**Total HC**	**Total FTE**	**Mean FTE PTP**
East Midlands	93.88	1765	1581.03	0.33	68.46	905	593.01	0.12	28.94	454	99.46	0.02	37.29	439	425.44	0.09
East of England	93.53	2209	1971.43	0.30	66.17	1153	733.53	0.11	30.38	584	139.48	0.02	36.09	499	477.71	0.07
Kent, Surrey and Sussex	93.65	1747	1375.23	0.28	73.41	1025	607.45	0.13	24.60	432	77.47	0.02	29.37	307	295.57	0.06
North Central and East London	94.64	1163	993.32	0.26	66.80	980	596.22	0.15	34.23	603	135.54	0.04	28.66	309	299.09	0.08
North East	93.71	1106	884.33	0.32	69.46	590	380.86	0.14	18.86	200	39.50	0.01	43.71	222	213.33	0.08
North West	85.49	2858	2442.93	0.31	60.96	1594	1057.13	0.14	25.75	776	166.45	0.02	35.58	677	647.84	0.08
North West London	92.42	747	647.96	0.26	63.20	605	371.71	0.15	32.87	318	86.20	0.04	19.10	124	121.92	0.05
South London	95.23	1093	933.68	0.25	76.63	1051	645.35	0.18	35.18	485	102.50	0.03	34.92	236	227.52	0.06
South West	94.81	2098	1712.33	0.34	81.64	1309	780.35	0.16	30.14	641	117.80	0.02	51.90	404	369.32	0.07
Thames Valley	93.31	912	759.86	0.29	82.01	596	368.99	0.14	33.05	219	56.87	0.02	46.86	192	183.95	0.07
Wessex	96.14	1153	1011.84	0.35	76.83	590	371.77	0.13	23.94	201	45.63	0.02	48.65	198	189.44	0.07
West Midlands	94.33	2316	2012.29	0.32	59.32	1206	779.62	0.13	31.11	693	137.64	0.02	38.29	498	471.25	0.08
Yorkshire and the Humber	90.18	2187	1793.51	0.31	65.03	1230	784.11	0.14	25.45	686	82.50	0.01	36.16	433	415.57	0.07
England	92.38	21 354	18 119.74	0.31	67.92	12 834	8070.10	0.14	28.66	6292	1287.04	0.02	36.78	4538	4337.95	0.07

FTE = full-time equivalent. HC = headcount. PTP = per thousand patients. ST = specialist trainee.

### GP partners

The proportion of practices with ≥1 GP partner varies between 85.49% (North West) and 96.14% (Wessex). The average FTE per thousand patients of partner GPs is higher in Wessex (0.35), South West (0.34), and East Midlands (0.33), while the regions with the fewest FTE per thousand patients are the three London regions: South London (0.25), North Central and East London (0.26), and North West London (0.26).

### Salaried GPs

Thames Valley has the highest percentage of practices with ≥1 salaried GP (82.01%), while West Midlands has the lowest at 59.32%. The regions with the largest FTE salaried GPs per thousand patients are the four London regions: South London (0.18), South West (0.16), North Central and East London (0.15), and North West London (0.15). The fewest salaried GP FTE per thousand patients are reported in East of England (0.11) and East Midlands (0.12).

### Locum and trainee GPs

North West London and North Central and East London practices report the highest locum GP employment rate in terms of FTE per thousand patients (0.04). North West London reports the lowest numbers of GPs in training (ST1-4 0.05 FTE per thousand patients) and has among the lowest combined FTE per thousand patients of partner and salaried GPs (0.41 FTE per thousand patients in each of North West London, North Central and East London, East of England, and Kent, Surrey and Sussex)

### Nurses

Table 3 presents workforce statistics for nurses and healthcare assistants in each region. A total of 93.57% of practices in England employ ≥1 practice nurse and 46.26% employ ≥1 advanced nurse. Employment of ≥1 practice nurse varies from 85.96% in North West London to 97.80% in South West. For advanced nurses, this ranges from 22.06% of practices in North Central and East London to 64.48% of practices in Wessex. Regions with the largest practice nurse FTE per thousand patients are South West (0.22), closely followed by Yorkshire and the Humber, East Midlands, and Wessex (0.21).The regions with the lowest practice nurse FTE per thousand patients are the three London regions: North West London (0.12), North Central and East London (0.12), and South London (0.15).

**Table 3.** Workforce statistics for nurses and healthcare assistants in each Health Education England region

**HEE Region**	**Practice nurse**				**Advanced nurse**				**Healthcare assistant**			
	**Practice with HC >0, %**	**Total HC**	**Total FTE**	**Mean FTE PTP**	**Practice with HC >0, %**	**Total HC**	**Total FTE**	**Mean FTE PTP**	**Practice with HC >0, %**	**Total HC**	**Total FTE**	**Mean FTE PTP**
East Midlands	95.18	1485	1007.49	0.21	57.33	621	475.24	0.10	82.93	884	613.66	0.13
East of England	95.49	1873	1201.96	0.18	55.94	750	567.79	0.09	77.74	995	661.62	0.10
Kent, Surrey and Sussex	95.24	1388	850.79	0.18	44.84	418	299.22	0.06	44.84	429	271.71	0.06
North Central and East London	91.13	755	465.39	0.12	22.06	129	85.81	0.02	54.43	369	257.47	0.07
North East	94.91	785	549.98	0.20	62.87	439	350.33	0.13	84.73	525	382.13	0.14
North West	91.67	2234	1516.96	0.20	41.76	794	624.46	0.08	66.29	1122	777.42	0.10
North West London	85.96	508	294.23	0.12	23.03	102	64.29	0.03	63.20	327	206.91	0.09
South London	92.46	814	528.65	0.15	29.90	171	115.91	0.03	60.55	338	225.52	0.07
South West	97.80	1782	1119.72	0.22	58.28	672	496.95	0.10	87.82	1063	682.83	0.14
Thames Valley	95.82	712	453.08	0.17	46.86	203	138.20	0.05	82.01	364	238.50	0.09
Wessex	96.14	947	598.55	0.21	64.48	389	285.98	0.10	87.64	463	308.65	0.11
West Midlands	93.83	1837	1189.96	0.19	43.32	600	448.25	0.07	73.05	958	630.58	0.10
Yorkshire and the Humber	92.71	1781	1219.73	0.21	54.46	823	636.67	0.11	81.85	1156	798.96	0.14
England	93.57	16 901	10 996.49	0.19	46.26	6111	4589.10	0.08	71.97	8993	6055.96	0.11

FTE = full-time equivalent. HC = headcount. PTP = per thousand patients.

### Direct patient care

Healthcare assistants (headcount total 8993) are the most numerous of the direct patient care categories (Table 3). Table 4 reports the remaining categories, with physiotherapists (headcount total 77), and physician associates (headcount total 213) having relatively low numbers.

**Table 4.** Workforce statistics for pharmacists, paramedics, physician associates, and physiotherapists in each Health Education England region

**HEE Region**	**Pharmacist**				**Paramedic**				**Physician associate**				**Physiotherapist**			
	**Practice with HC >0, %**	**Total HC**	**Total FTE**	**Mean FTE PTP**	**Practice with HC >0, %**	**Total HC**	**Total FTE**	**Mean FTE PTP**	**Practice with HC >0, %**	**Total HC**	**Total FTE**	**Mean FTE PTP**	**Practice with HC >0, %**	**Total HC**	**Total FTE**	**Mean FTE PTP**
East Midlands	18.92	115	82.07	0.02	3.53	28	26.15	0.01	0.93	6	4.31	0.00	0.56	3	1.97	0.00
East of England	17.14	145	102.32	0.02	9.17	89	81.35	0.01	3.61	34	30.31	0.01	1.05	7	2.84	0.00
Kent, Surrey and Sussex	15.28	89	66.30	0.01	20.44	159	134.69	0.03	2.58	14	11.92	0.00	0.60	4	1.92	0.00
North Central and East London	11.75	72	45.90	0.01	0.41	4	2.03	0.00	2.89	20	18.25	0.01	0.21	2	0.29	0.00
North East	19.16	72	43.48	0.02	1.80	6	5.92	0.00	0.30	1	1.00	0.00	0.90	3	1.59	0.00
North West	14.70	206	139.87	0.02	1.12	13	11.55	0.00	1.87	28	26.84	0.00	0.75	8	2.76	0.00
North West London	16.29	69	43.65	0.02	0.00	0	0.00	0.00	1.69	8	5.87	0.00	0.56	3	1.01	0.00
South London	18.09	82	58.47	0.02	2.51	11	11.05	0.00	4.02	24	23.11	0.01	0.00	0	0.00	0.00
South West	21.76	131	82.32	0.02	13.97	101	77.02	0.02	0.60	3	2.09	0.00	2.40	21	10.56	0.00
Thames Valley	26.36	85	53.23	0.02	15.90	60	49.97	0.02	4.60	13	10.16	0.00	2.09	7	2.90	0.00
Wessex	11.20	39	27.64	0.01	7.34	28	25.81	0.01	0.39	1	1.00	0.00	1.16	3	1.32	0.00
West Midlands	18.64	184	110.36	0.02	2.64	30	25.88	0.00	3.15	31	25.98	0.00	0.88	7	3.44	0.00
Yorkshire and the Humber	22.62	210	134.78	0.02	3.13	25	22.39	0.00	2.83	30	26.97	0.01	1.34	9	5.52	0.00
England	17.64	1499	990.39	0.02	5.61	554	473..81	0.01	2.32	213	187.81	0.00	0.92	77	36.12	0.00

FTE = full-time equivalent. HC = headcount. PTP = per thousand patients.

## DISCUSSION

### Summary

This analysis demonstrates regional variation in both the practitioner composition of the workforce and the total primary care practitioner workforce in terms of FTE employment per thousand patients.

Regional variation in practice list size may be associated with specific aspects workforce composition. For example, the largest average list sizes are in Wessex, which also has the highest proportion of practices with ≥1 GP partner and ≥1 practice nurse. Conversely, as the region with smallest average list sizes, North West records the fewest practices as having ≥1 partner GP and ≥1 practice nurse.

Three of the four lowest average total workforce ratios (in terms of FTE per thousand patients) are in London regions. They also have the fewest FTE per thousand patients of GP partners and the highest FTE per thousand patients of salaried GPs. This is of particular interest because workforce participation data (that is, FTE) indicates that on average GP partners are working more hours per week than salaried GPs. This is consistent with other studies,^23^ and has potential implications for achieving the benefits associated with continuity of patient care.^35^

Locum GP employment is highest in London regions, while the distribution of trainee GPs (those specialist trainees in years 1–4 of GP training programmes) is dispersed across HEE regions. These data do not differentiate between trainees in early and later stages of their training, therefore it is not possible to determine the extent to which they require supervision or when they will be qualified to work independently.

Practice nurse and advanced nursing ratios are low in London regions, while higher ratios of advanced nurse practitioners tend to occur in regions that also have higher ratios of healthcare assistants (highest in South West) and higher average total workforce ratios. Employment of advanced level nurses shows marked regional variation with an FTE ratio per thousand patients of 0.13 in the North East compared with a ratio of 0.02 in North Central and East London. Regional availability of the training and support to prepare for advanced nursing roles may account for variation in their employment in different HEE regions, but deeper investigation is needed.

Apart from healthcare assistants, other staff in direct patient care categories are reported relatively infrequently and at levels that are not suitable for comparable analyses. This highlights the limited contribution to health care made by these practitioners. It also demonstrates the scale of expansion in numbers that would be required for them to provide a meaningful volume of potential ‘substitutes’ for GPs or to have an impact on GP workload and access for patients by performing complementary tasks. Furthermore, it is unclear whether these practitioners reduce GP work by effective substitution or whether their supervision may generate additional GP work.^36^

### Strengths and limitations

This study used an established and widely referenced national (England) dataset, which is updated, monitored, and checked by NHS Digital. The dataset reports greater detail than ever before in terms of FTE working and multiple role descriptions.

New reporting processes have replaced older processes to improve data reporting. Guidance updates are regularly distributed by NHS Digital and a quarterly data refresh means that practice staff become familiar with the process.

In common with self-reporting processes generally, there are deficiencies in data quality. Some GP practices do not submit regular or full returns, therefore, the dataset is incomplete and some practices have been dropped from this analysis. Furthermore, it is not certain that data are captured for all practitioners who are not directly employed by or wholly based at GP practices, in other words, those who may be regarded as employed by an external organisation such as a clinical commissioning group, or who work at multiple sites.

It is important to recognise that these workforce data do not add information about the roles, duties, or responsibilities undertaken by practitioners and therefore cannot add detailed information about how they contribute to delivery of health care.

It was also observed that the practitioner descriptors guidance supplied by NHS Digital^34^ are open to interpretation and do not always match the role titles used in GP practices. Furthermore, since practices can only record one role for each staff member, those having more than one role cannot be recognised in the dataset, and their additional roles may be under-reported.

The analysis has not been extended to include contextual factors, such as variation in demographic characteristics, or the prevalence of illnesses that may be associated with different health needs in different regions or practice populations.

### Comparison with existing literature

No published analyses were found about the distribution of multiple types of practitioners, apart from online national summaries from NHS Digital. In contrast with previous studies reporting geographical variation in the distribution of GPs and practice nurses,^32^^,^^33^ reports from HEE and the King’s Fund refer to regional variation but do not report in sufficient depth to reveal regional differences in the composition of the workforce.^31^^,^^37^

### Implications for research and practice

This article sets out a methodological approach to understanding variation in workforce composition and establishes a baseline for comparison with future datasets. Regional level statistics are presented on the current scale of skill mix employment in primary care and regional variation is indicated in the different types of practitioner in terms of FTE per thousand patients. This provides a more nuanced picture than has previously been available and lays the foundations for future analysis of other data that are potentially associated with workforce capacity and practitioner composition. This is an essential step towards a broader consideration of what sort of workforce is required to meet local health needs and the resources needed for their training and continuing deployment.^16^

In addition to monitoring how the workforce evolves over time, this article provides a foundation for future analyses, including overall costs and patient outcomes, which will usefully inform policy development with regard to workforce planning, practitioner training, and commissioning services. Additional work is needed to identify changes in primary care delivery that cannot be captured solely through analysis of workforce data; for example, the extent to which tasks and responsibilities are transferred between practitioners or whether additional work is generated for GPs by multi-level supervision of less qualified practitioners.

The extent to which newer types of practitioner can substitute for a depleted GP workforce remains unclear and consequences for costs have not yet been fully evaluated. Significant time and investment in training will be needed if the small proportion of newer types of practitioner is to expand sufficiently to relieve pressure on existing primary service providers. Therefore, delays in achieving widespread skill mix change cannot deliver a full and immediate solution to the current GP UK workforce crisis.
